# Upregulation of IL-15 in the placenta alters trophoblasts behavior contributing to gestational diabetes mellitus

**DOI:** 10.1186/s13578-021-00533-4

**Published:** 2021-02-08

**Authors:** Jiaqi Li, Yuan Li, Xuan Zhou, Lijie Wei, Jingyi Zhang, Shenglan Zhu, Huiting Zhang, Xuan Gao, Lali Mwamaka Sharifu, Shaoshuai Wang, Ling Xi, Ling Feng

**Affiliations:** National Clinical Research Center of Gynecology and Obstetrics, Tongji Hospital, Tongji Medical College, Huazhong University of Science and Technology, 1095 Jiefang Anv, Wuhan, Hubei, 430030 China

**Keywords:** IL-15, Placenta, Gestational diabetes mellitus, Trophoblasts, JAK/STAT signaling pathway

## Abstract

**Background:**

Interleukin-15 (IL-15), a member of the ‘four α-helix bundle’ cytokine family, has been associated with many inflammatory and metabolic diseases. Abnormal expression of IL-15 has been linked to the occurrence and development of obesity and diabetes. However, there is a paucity of research on the involvement of IL-15 in Gestational Diabetes Mellitus (GDM). This study aims at investigating the role of IL-15 in the pathogenesis of GDM.

**Results:**

IL-15 was consistently expressed in the placenta throughout pregnancy and dynamically changed with pregnancy progress. Trophoblasts have been identified as the major source of IL-15 in the placenta. Expression of IL-15 was significantly increased in the placenta of GDM and in the trophoblasts cultured with high glucose (HG). In our study, expression of IL-15 in the placenta was positively correlated with blood glucose concentration of 75 g Oral Glucose Tolerance Test (OGTT), and was inversely correlated with weight of newborns. Further investigations in vitro showed that exogenous addition of IL-15 promoted trophoblasts proliferation, improved invasion and tube formation ability by activating the JAK/STAT signaling pathway, which be blocked by JAK inhibitors.

**Conclusion:**

Our results demonstrated that IL-15 expression in the placenta was dynamically changing during pregnancy, and it was upregulated in the placenta of GDM patients. Furthermore, IL-15 altered the biological behavior of trophoblasts through JAK/STAT signaling pathway in vitro, and may contributed to the placental pathology of GDM. Our findings provide a new direction for studying the pathophysiological changes of placenta in GDM.

## Introduction

Gestational Diabetes Mellitus (GDM) is defined as “glucose intolerance with onset or first recognition during pregnancy”. It is the most common metabolic complication during pregnancy. In recent years, the prevalence of GDM has increased rapidly reaching approximately 15% in China [1]. GDM can lead to many other complications, which has a major impact on both the maternal and fetal health outcomes. In the maternal case, there is an increased risk of pre-eclampsia during the pregnancy and Type 2 Diabetes Mellitus (T2DM) after delivery. In fetus and newborns, GDM leads to an increased risk of stillbirth, macrosomia, growth restriction, and predisposition to T2DM and obesity [2]. To date, the pathogenesis of GDM has not been fully elucidated.

To maintain a successful embryo implantation and fetal growth in a normal pregnancy, the maternal body has to be in a state of anti-inflammatory and pro-inflammatory balance for an extended period of time [3]. However, this balance is broken in GDM, since pro-inflammatory cytokines in the placenta such as CRP, IL-1β, IL-6, IL-8 and TNF-α are significantly increased and anti-inflammatory cytokines such as IL- 4 and IL-10 are significantly decreased [4]. On the one hand, this pro-inflammatory state resulting from GDM leads to the adaptive changes of structure in the placenta, which manifests as enlarged, thick, and plethoric placenta with high chorionic villi and networks of blood vessels [5]. On the other hand, these pro-inflammatory cytokines secreted by the placenta bind to their corresponding receptors leading to the activation of downstream signaling pathways including: Nuclear Factor-κB (NF-κB), Peroxidase Proliferator-Activated Receptor (PPAR), AMP-Activated Protein Kinase (AMPK), Glycogen Synthase Kinase 3 (GSK3), PI3K/mTOR, inflammatory corpuscles and Endoplasmic Reticulum Stress (ERS) [6, 7]. Subsequently, a series of biological effects of trophoblasts are induced including: proliferation and invasion, reduction of the activity of insulin and glucose receptors[8], and the secretion of pro-inflammatory factors, which further affects the maternal metabolism of glucose and lipids and contributes to the pathogenesis of GDM itself [9]. Thus, GDM is also called a chronic low-grade inflammatory disease [10].

Recently, Interleukin-15 (IL-15), an inflammatory cytokine which belongs to the “four α-helix bundle” cytokine family, has received great attention [11]. IL-15 expression was significantly increased in the peripheral blood of patients with type 1 diabetes, and positively correlated with the concentration of glycosylated hemoglobin [12]. What’s more, the increased IL-15 expression in combination with other inflammatory factors in the peripheral blood acts as a molecular marker for T2DM [13]. In an obese rat model, IL-15 from the skeletal muscles was an anti-inflammatory cytokine, which can regulate lipid deposition and mobilization, improve Brown Adipose Tissue (BAT) function and mitochondrial activity, and ultimately reduce the adipose tissue mass [14]. Mechanistically, although the mRNA of IL-15 can be found in many cells and tissues including: adipose, which is the most important source of inflammatory factors during pregnancy. IL-15 protein can only be expressed by certain cell types due to a strict post-transcriptional regulation, such as skeletal muscles [15, 16]. By binding the receptor of IL-15 (IL-15R) and forming a membrane-bound IL-15-IL-15R complex, IL-15 mainly phosphorylates the JAK (Janus Kinase)/STAT (Signal Transducer and Activator of Transcription proteins) complex, initiates the signal transduction and then induces expression and activation of downstream effector molecules [17]. In many metabolic diseases, JAK/STAT signaling pathway activation was also confirmed and implicated in a range of molecular processes such as inhibition of gluconeogenesis and decrease of insulin sensitivity [18]. Inspired by the important roles of IL-15 in metabolic diseases, we are curious whether IL-15 is also involved in the pathophysiological process of GDM.

In this study, we first measured the expression of IL-15 in the placenta at the different periods of pregnancy and identified the trophoblast cells as a major source of IL-15. Subsequently, we used samples from GDM patients, normoglycemic pregnant women and also from cell models cultured with high glucose (HG) to investigate changes in IL-15 expression. Finally, we administered IL-15 in vitro to determine its effects on biological functions of trophoblasts and made preliminary exploration on related mechanisms. Findings from this study provide new insights into the pathophysiological of GDM.

## Results

### IL-15 was expressed in the placenta and trophoblast cell lines

**Fig. 1:**
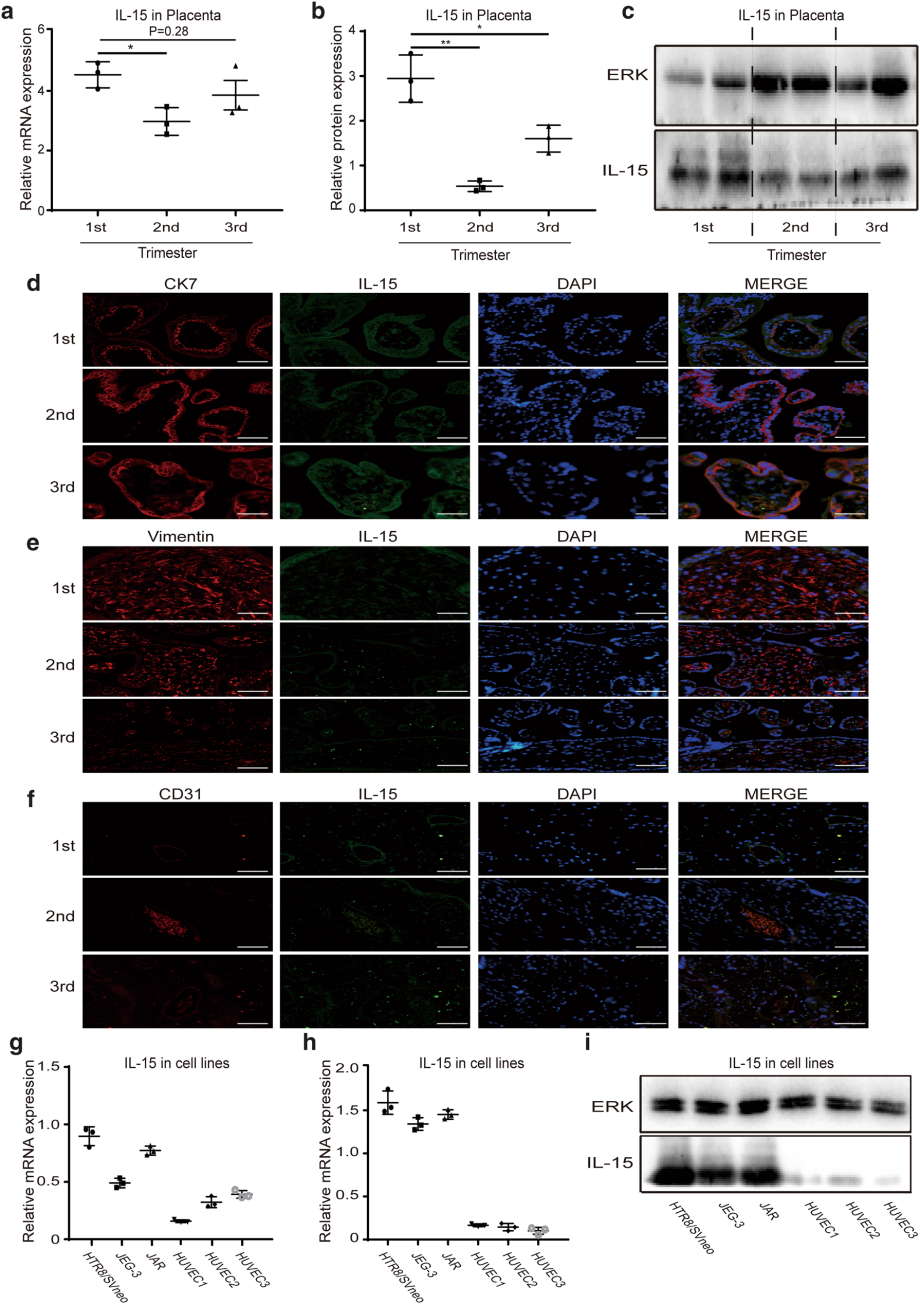
IL-15 expression in the placenta of different trimesters and trophoblast cell lines. **a** IL-15 mRNA level in human placental samples from different trimester as determined by RT-PCR. First trimester: 1st (n = 3), Second trimester: 2nd (n = 3), and Third trimester: 3rd (n = 3). **b**, **c** Relative expression of IL-15 protein in placenta tissues from different trimesters and representative images as measured by western blot. **d** Immune-fluorescence double staining of IL-15 (green) and CK7 (red) in placental samples of different trimesters. **e** Immune-fluorescence double staining of IL-15 (green) and vimentin (red) in placental samples of different trimesters. **f** Immune-fluorescence double staining of IL-15 (green) and CD31 (red) in placental samples of different trimesters. For d-f, bar = 50 μm. **g** IL-15 mRNA expression in trophoblast cell lines (HTR-8/SVneo, JEG-3, JAR) and HUVEC (n = 3). **h**, **i** IL-15 protein expression in trophoblast cell lines and HUVEC and representative images as determined by western blot. *P < 0.05, ** P < 0.01

To investigate whether IL-15 was expressed in the placenta, we measured the expression of IL-15 in the placental samples from different trimester. Our results showed that the level of IL-15 mRNA was highest in the first trimester placenta, then it reduced significantly in the second trimester placenta, and increased again in the third trimester. IL-15 mRNA expression was normalized to the expression of β-actin housekeeping gene (Fig. 1a). Similar to the gene expression levels, western blot analysis showed that IL-15 protein was consistently expressed in the placenta of different trimester. The level of IL-15 protein was the highest in the first trimester and was the lowest in the second trimester, then it increased again in the third trimester. ERK protein was used as the internal reference (Fig. 1b, c).

Next, co-localization by immunofluorescence was used to identify the source of IL-15 in the placenta. Trophoblasts, marked by CK7 (red) antibody, were strongly positive for the IL-15 (green) antibody staining in all sections (Fig. 1d). We observed no staining of IL-15 (green) antibody in interstitial cells marked by Vimentin (red) in villous, but similar nonspecific staining was observed in all sections (Fig. 1e). Vascular endothelial cells, marked by CD31 (red), stained slightly with IL-15 (green) antibody in all sections (Fig. 1f). In summary, trophoblasts were the major source of IL-15 in the placenta throughout the pregnancy.

To further confirm the results of immunofluorescence, we measured the expression of IL-15 in three types of trophoblast cell lines (HTR-8/SVneo, JEG-3 and JAR) and in three primary Human Umbilical Vein Endothelial Cell (HUVEC), which isolated from three normal pregnant women using collagenase digestion of umbilical vein [19]. IL-15 mRNA expression was detected in all these cell types, but its level in HUVEC was much lower than that in trophoblast cell lines (Fig. 1g). IL-15 protein expression was consistent in the result of mRNA, which was much lower in HUVEC than trophoblast cell lines (Fig. 1h, i).

### Clinical characteristics of patients and newborns

The clinical characteristics of patients and newborns in this study were presented in Table 1. GDM group and normoglycemic group had similar maternal age and height. There was no significant difference in the pre-pregnant BMI and delivery BMI between two groups. As expected, compared to normoglycemic group, the blood glucose levels of the OGTT and fasting blood glucose level before delivery in GDM group were both much higher. Gestational week at delivery in GDM group was significantly lower than in the normoglycemic group. Systolic and diastolic blood pressure at late gestation were not significantly different in two groups. Newborns parameters including fetal sex, fetal weight and placental weight did not differ significantly between the normoglycemic group and GDM group.

**Table 1:** Clinical characteristics of patients and newborns

	Normoglycemic group(n = 20)	GDM group (n = 20)
Maternal variables		
Age (years)	31.7 ± 1.284 (24–43)	33.2 ± 1.102 (26–43)
Height (m)	1.609 ± 0.01 (1.55–1.73)	1.592 ± 0.01 (1.5–1.67)
Pre-pregnancy weight (kg)	56.28 ± 1.471 (45–68.5)	54.18 ± 1.437 (45–65)
Pre-pregnancy BMI (kg/m^2^)	21.79 ± 0.6428 (17.625–27.038)	21.4 ± 0.5599 (17.928–28)
Delivery weight (kg)	70.4 ± 1.325(61–80)	67.5 ± 1.978(51.5–85)
Delivery BMI (kg/m^2^)	27.25 ± 0.5743 (22.862–32.466)	26.65 ± 0.7427 (21.4–31.992)
Fasting blood glucose before delivery (mmol/l)	4.561 ± 0.1175 (3.78–5.31)	5.586 ± 0.1853 (4.23–6.34)***
OGTT at mid gestation (mmol/l)		
0 h	4.623 ± 0.0604 (3.86–5)	5.244 ± 0.1125 (4.22–6.21)***
1 h	8.31 ± 0.2403 (5.94–9.8)	10.41 ± 0.4141 (7.49–13.04)***
2 h	7.288 ± 0.1757 (5.41–8.44)	9.194 ± 0.3636 (6.44–13.42)***
Gestational age at delivery (weeks)	39.5 ± 0.1866(38.2–41)	38.5 ± 0.3258 (34–41.2)*
Systolic blood pressure (mmHg)	124 ± 2.237 (106–138)	127 ± 1.752 (108–138)
Diastolic blood pressure (mmHg)	74 ± 1.338 (62–86)	77 ± 1.477 (66–89)
Newborn variables		
Fetal sex (%male)	55%	45%
Fetal weight (g)	3492 ± 71.94 (2900–4100)	3598 ± 99.24 (2450–4300)
Placental weight (g)	553.3 ± 6.789 (520–665)	574.5 ± 5.501 (525–680)

Data are presented as mean ± SEM (range). All pregnancies were singleton, normotensive, nonsmoking, not alcohol or drug consuming, and without intrauterine infection or any other medical or obstetrical complications except GDM. OGTT glucose measure was 2-h post glucose challenge (75 g). *P < 0.05 vs normal. ***P < 0.001 vs normal

### Up-regulation of IL-15 expression in the placenta of GDM and in trophoblasts cultured with HG

**Fig. 2:**
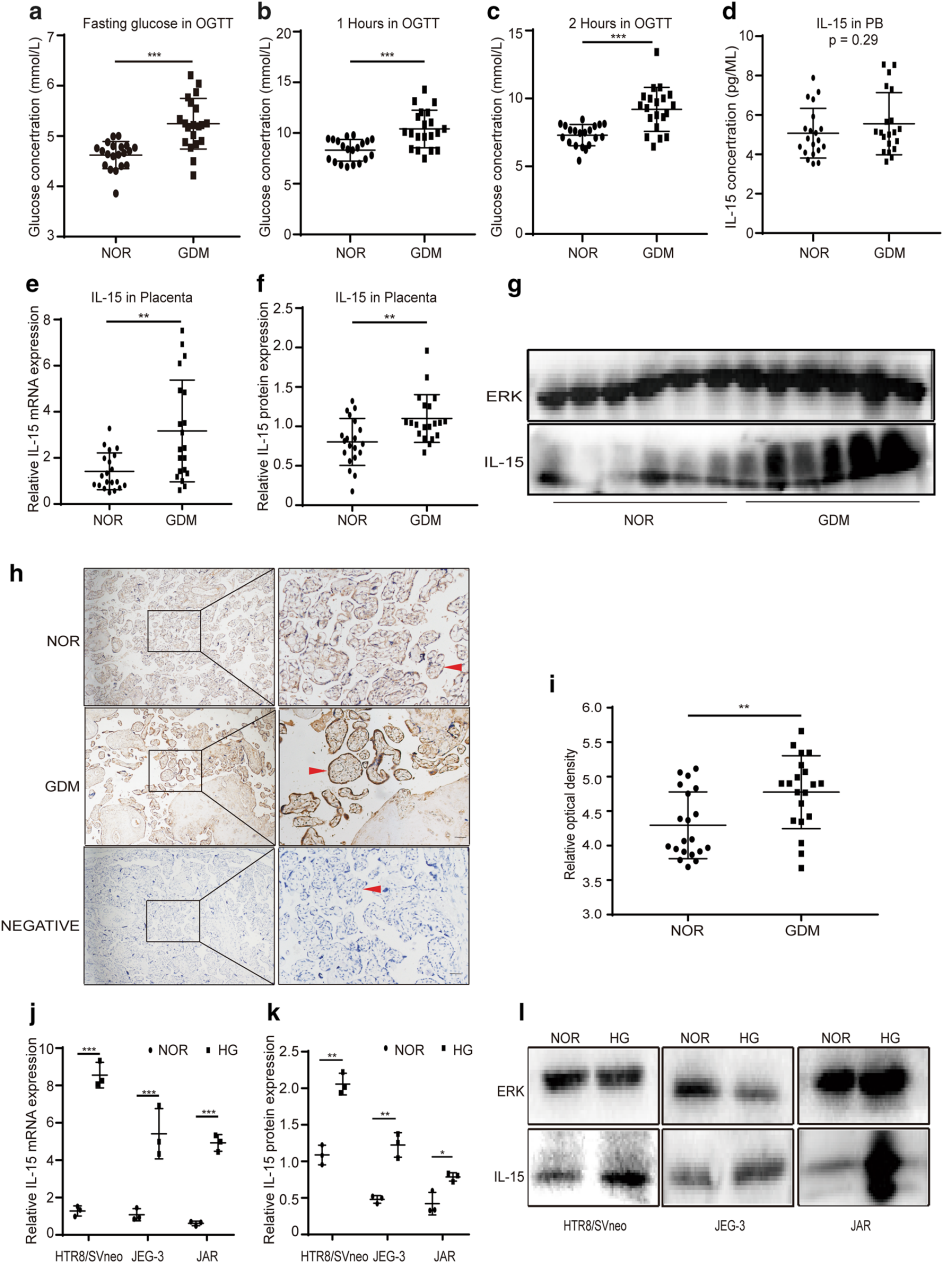
IL-15 expression increased in the placenta of GDM and in trophoblasts cultured with HG*.* **a** Fasting glucose in OGTT of participants. **b** Postprandial 1 h glucose (1 h) in OGTT of participants.** c** Postprandial 2 h glucose (2 h) in OGTT of participants. **d** IL-15 level in peripheral blood (PB) of patients. **e** RT-PCR detected expression of IL-15 mRNA in the placenta of participants. **f**, **g** Expression of IL-15 protein and representative images of western blot in the placenta of participants. **h**, **i** Representative Immunohistochemistry images and the relative optical density of IL-15 protein in the placenta of participants. Red arrows indicate placental villous. Bar = 25 μm. **j** The expression of IL-15 mRNA in trophoblast cell lines (HTR8/SVneo, JEG-3, JAR) in HG (25 mmol/L) as measured by RT-PCR. **k**, **l** The expression of IL-15 protein in trophoblast cell lines in HG detected by western blot and representative images of it. *P < 0.05, **P < 0.01, ***P < 0.001

The expression of IL-15 in the peripheral blood and the placenta of 20 GDM patients and 20 normoglycemic pregnant women were measured to evaluate whether IL-15 was involved in the pathogenesis of GDM. In the peripheral blood, the blood glucose levels at three times in the OGTT of GDM group were all significantly higher than in normoglycemic group (Fig. 2a–c). The level of IL-15 in normoglycemic group was slightly lower than GDM group, but the difference was not significant (Fig. 2d).

Subsequently, we measured the expression of IL-15 mRNA and protein in the placenta. IL-15 mRNA in GDM group was much higher than normoglycemic group (Fig. 2e). IL-15 protein expression was also significantly higher in GDM group (Fig. 2f, g), which was consistent in the result of immunohistochemistry analysis (Fig. 2h, i).

In order to further identify whether the expression of IL-15 in trophoblasts influenced by HG in vitro, we cultured the trophoblasts in a high glucose medium. The results showed that compared to normal glucose group, the expression of IL-15 mRNA in all trophoblast cell lines increased remarkably in HG group (Fig. 2j). The level of IL-15 protein also increased significantly in HG group (Fig. 2k, l). These results suggest that HG promoted trophoblasts to express more IL-15 protein both in vivo and in vitro.

### Correlation between expression level of IL-15 in the placenta and clinical characteristics

**Fig. 3:**
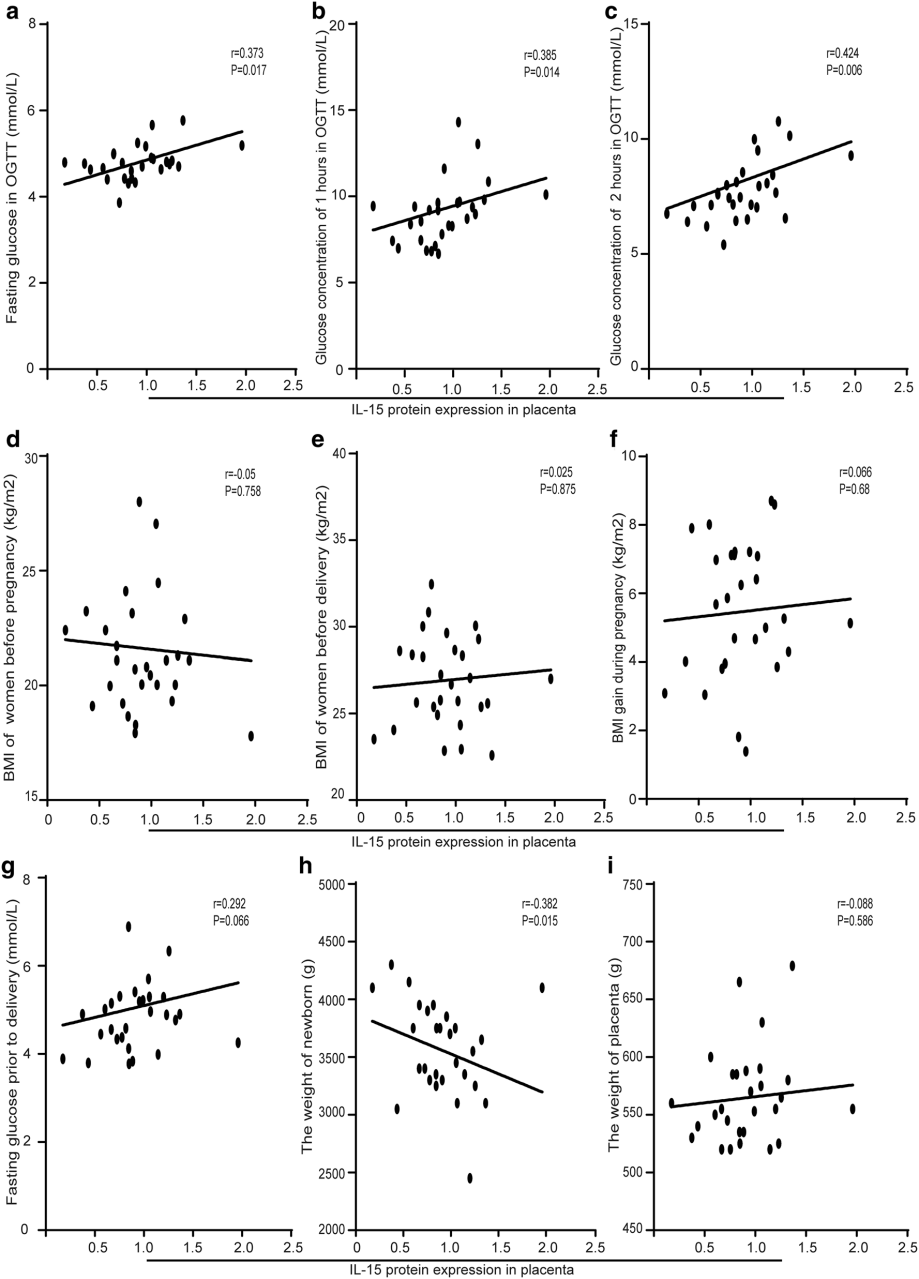
Correlations between expression level of IL-15 in the placenta and clinical characteristics. **a–c** The correlation between expression level of IL-15 in the placenta and glucose concentrations in OGTT of participants. **d-f** The correlation between expression level of IL-15 in the placenta and BMI of participants in different periods. **g** The correlation between expression level of IL-15 in the placenta and fasting glucose level of participants before delivery. **h** The correlation between expression level of IL-15 in the placenta and weight of newborns. **i** The correlation between expression level of IL-15 in the placenta and weight of placenta. *P < 0.05, **P < 0.01, ***P < 0.001

Correlation analysis with the spearman test was used to study relations between expression level of IL-15 in the placenta and clinical characteristics of the participants. A positive correlation was established between expression level of IL-15 in the placenta and blood glucose level at three time points in the OGTT (Fig. 3a–c). No correlation was observed between expression level of IL-15 in the placenta and BMI of the participants including: BMI before pregnancy (Fig. 3d), BMI before delivery (Fig. 3e), and gain of BMI during pregnancy (Fig. 3f). Also, there was no correlation between IL-15 expression and fasting blood glucose level prior to delivery (Fig. 3g). The expression level of IL-15 in the placenta has a negative correlation with the weight of newborns (Fig. 3h), whereas has no correlation with placenta weight (Fig. 3i).

### IL-15 promoted trophoblasts proliferation in vitro

**Fig. 4:**
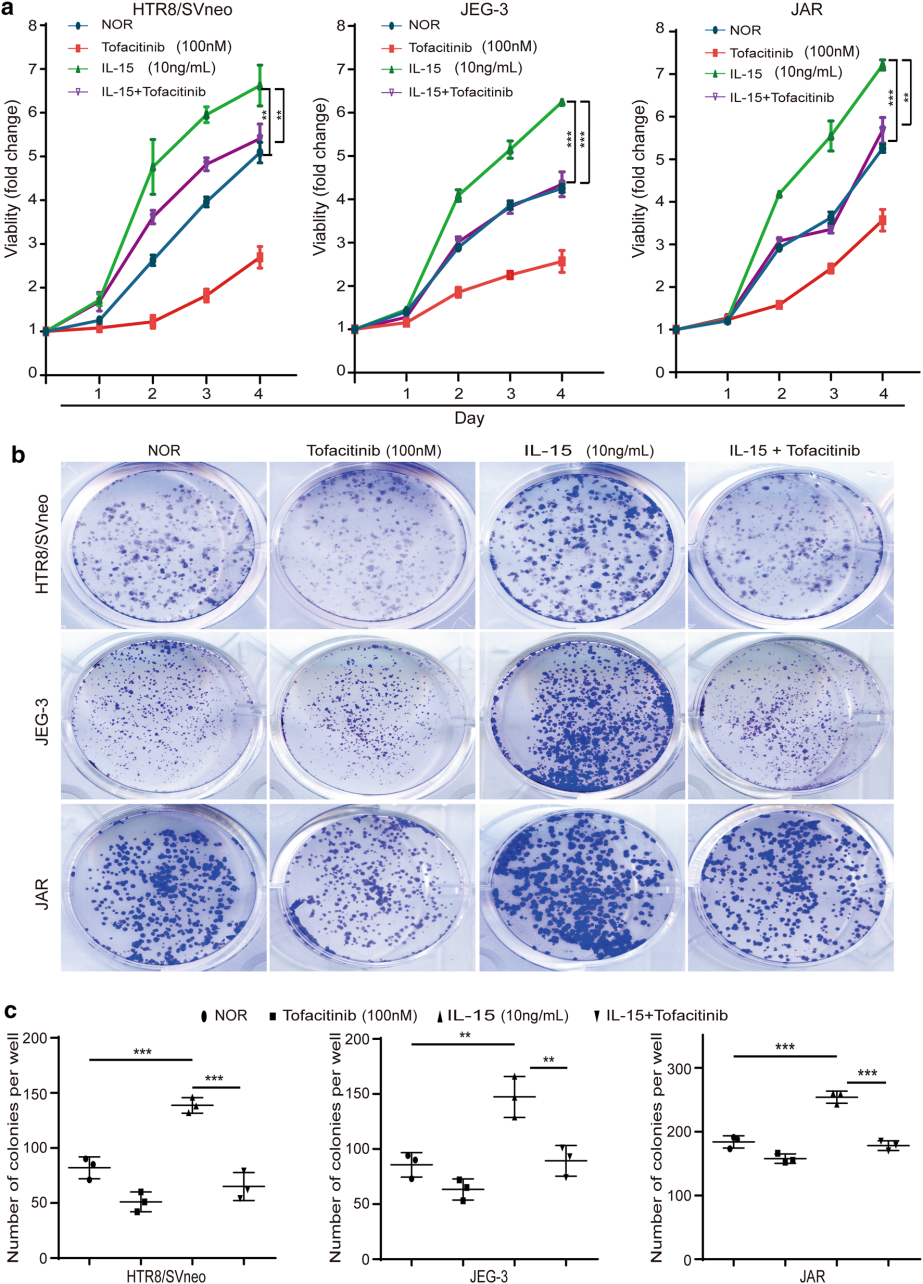
IL-15 promoted trophoblasts proliferation in vitro. **a** The proliferation of HTR8/SVneo, JEG-3 and JAR in different groups was measured using CCK-8 assay. **b**, **c** Representative images of colony formation and mean number of colonies formed of HTR8/SVneo, JEG-3 and JAR in different groups. **P < 0.01, ***P < 0.001

To study whether and how IL-15 participates in the pathological changes of placenta in GDM, we performed the CCK‐8 assay and colony forming assay to determine the effect of IL-15 on trophoblasts proliferation ability. All trophoblast cell lines were divided into four groups according to the culture medium components including: IL-15 group, Tofacitinib group (a JAK inhibitor), IL-15+ Tofacitinib group and normal group. In the CCK8 assay, we found that proliferation of trophoblasts in the IL-15 group was significantly increased compared to the normal group, whereas in IL-15+ Tofacitinib group was decreased remarkably compared to the IL-15 group (Fig. 4a). In the result of colony forming assay, the number of colonies per well in the IL-15 group was much more than the normal group in all trophoblast cell lines. And the number of colonies in IL-15+ Tofacitinib group were much lower than in IL-15 group in all trophoblast cell lines (Fig. 4b, c). In summary, we found that exogenous application of IL-15 can significantly promote trophoblasts proliferation, which can be reversed by JAK inhibitors in vitro.

### IL-15 promoted trophoblasts invasion and tube formation in vitro

**Fig. 5:**
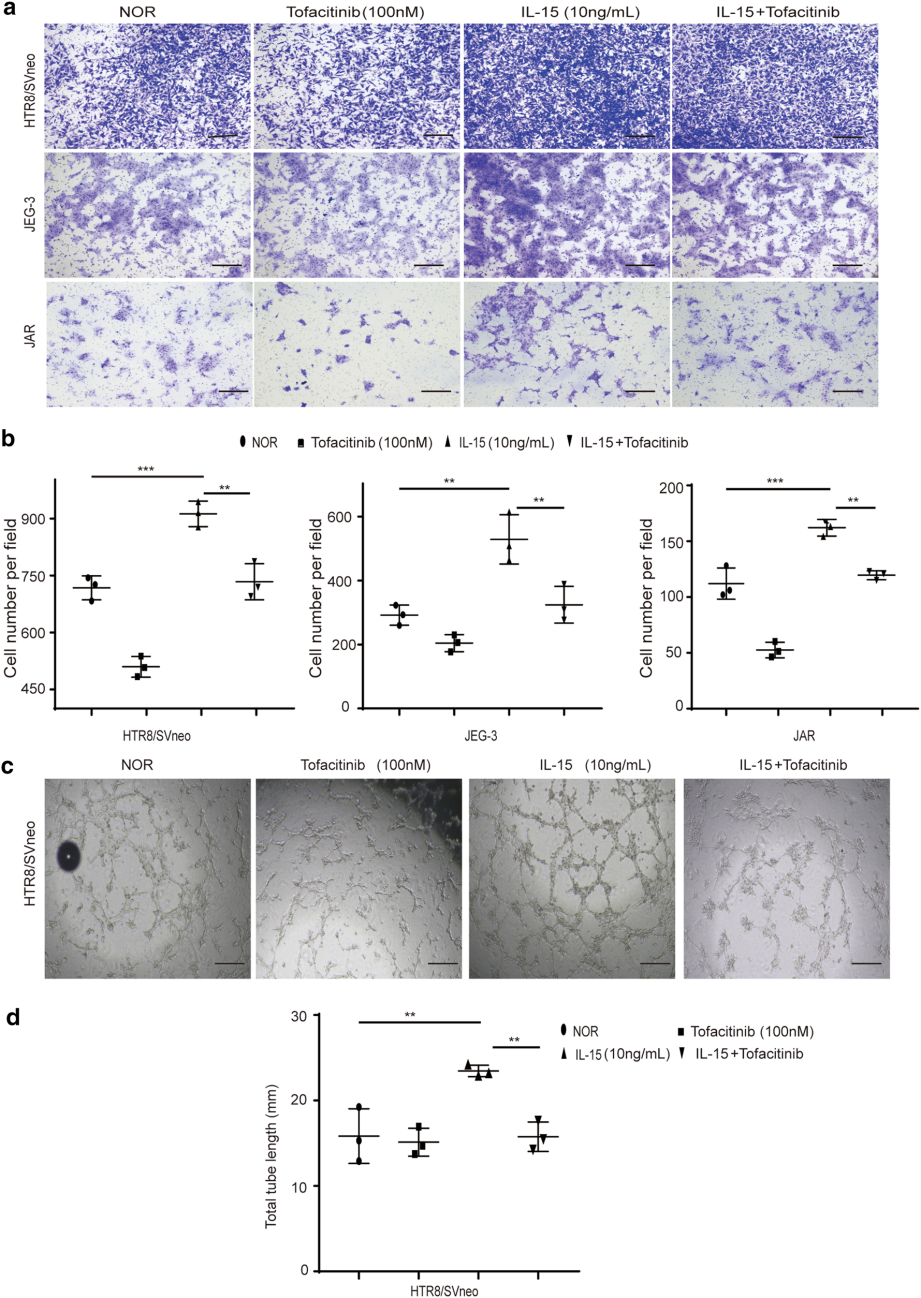
IL-15 promoted trophoblasts invasion and tube formation. **a**, **b** Representative images of invasion and mean number of invasion cells of HTR8/SVneo, JEG-3 and JAR in different groups as detected by a transwell invasion assay. Bar = 50um. **c**, **d** Representative images of tube formation and mean number of total tube length of HTR8/SVneo in different groups as measured by the tube formation assay. Bar = 100um. *P < 0.05, **P < 0.01, ***P < 0.001

In order to determine the effect of IL-15 on trophoblasts ability of invasion and tube formation, we performed the Transwell invasion assay and tube formation assay. The result of Transwell invasion assay showed that cell numbers in the IL-15 group were significantly higher than in the normal group in all trophoblast cell lines. In the IL-15+ Tofacitinib group, the number of cells were much lower than the IL-15 group in all trophoblast cell lines (Fig. 5a, b).

HTR-8/SVneo is a type of extravillous trophoblast cells, which has the tube formation ability [20]. Our results showed that the total tube length of cells in the IL-15 group was significantly higher than the normal group, whereas in the IL-15+ Tofacitinib group was significantly lower than the IL-15 group (Fig. 5c, d). In summary, we found that IL-15 increased the trophoblasts’ ability of invasion and tube formation, which also be reversed using JAK inhibitors in vitro.

### IL-15 activated JAK/STAT signaling pathway in trophoblasts in vitro

**Fig. 6:**
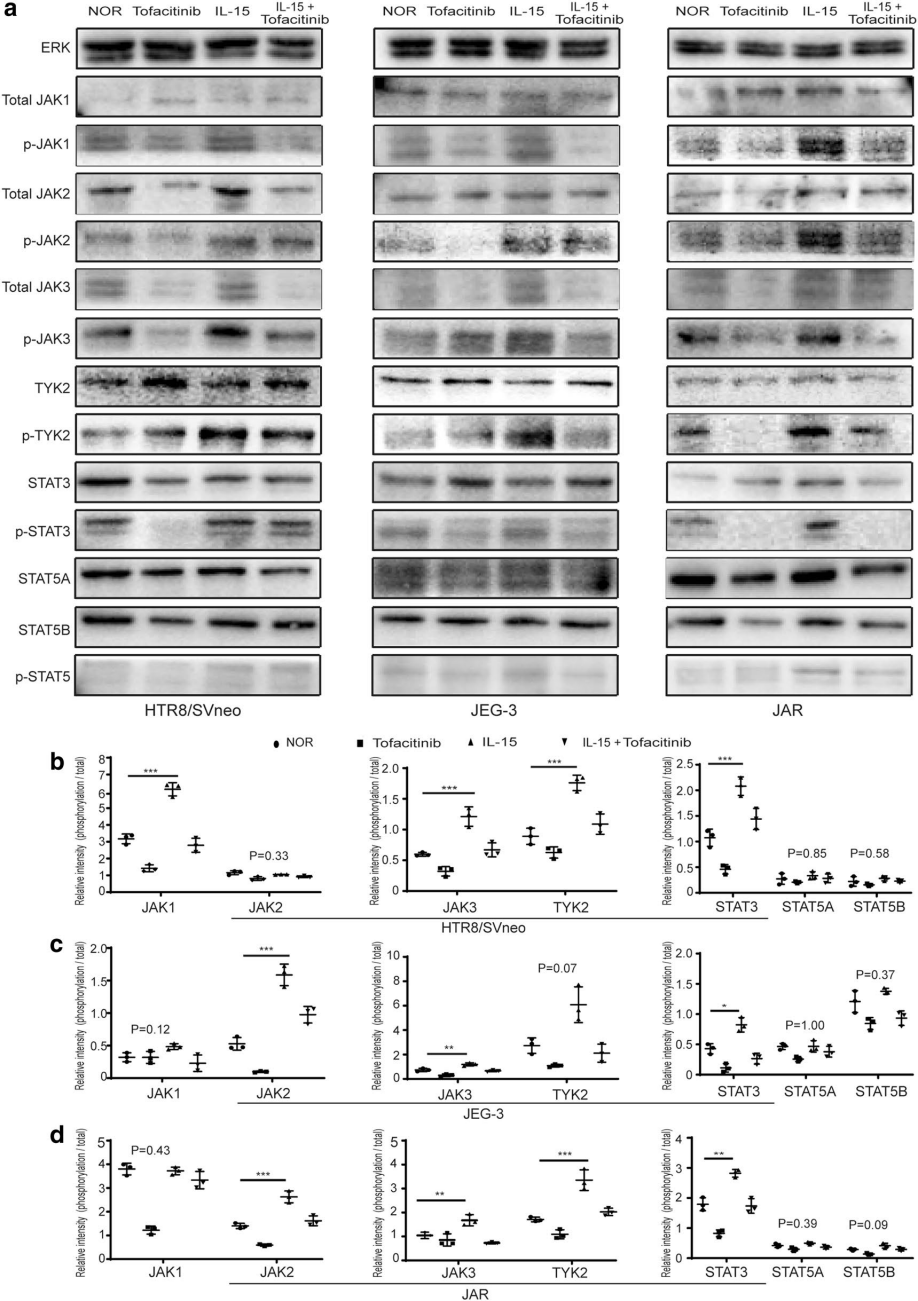
IL-15 activated the JAK/STAT signaling pathway in trophoblasts in vitro. **a** Representative of western blot showed the expression of proteins associated with JAK/STAT signaling pathways in different groups of HTR8/SVneo, JEG-3 and JAR. **b**–**d** The relative expression of phosphorylation/ total protein in JAK/STAT signaling pathways in different groups of HTR8/SVneo, JEG-3 and JAR. *P < 0.05, **P < 0.01, ***P < 0.001

Previous studies indicate that IL-15 mainly utilizes the JAK/STAT signaling pathway to initiate signal transduction for cellular activation [17]. To further study the mechanism of IL-15 in trophoblasts, we detected the phosphorylation of JAK/STAT signaling pathway-related proteins in trophoblasts. The results showed that the expression level of phosphorylation/total protein in JAK/STAT signaling pathway-related proteins in the IL-15 group significantly higher than the normal group in all trophoblast cell lines. The level of phosphorylated/total protein with JAK1, JAK3, TYK2 and STAT3 in HTR8/SVneo increased significantly in the IL-15 group compared to normal group (Fig. 6a, b). In JEG-3, phosphorylated/total protein of JAK2, JAK3 and STAT3 were significantly up-regulated in the IL-15 group compared to normal group (Fig. 6a, c). In JAR, phosphorylated/total protein of JAK2, JAK3, TYK2 and STAT3 were significantly up-regulated in the IL-15 group compared to normal group (Fig. 6a, d). In summary, we show that exogenous application of IL-15 in trophoblast cell lines activated the JAK/STAT signaling pathway to alter the biological activity of trophoblasts.

## Discussion

In this study, we first determined that the placenta from different trimesters continuously secreted IL-15, and the level of IL-15 changed in different trimesters. IL-15 expression was demonstrated to be highest in the first trimester placenta, declined in the second trimester placenta and increased in the third trimester, which was also observed in the amniotic fluid [21]. This expression pattern of IL-15 was similar to that of many pro-inflammatory factors in the placenta such as IL-8, TNF-α, MCP-1, etc., which may adapt to the maintenance of normal pregnancy [3, 22]. In the early pregnancy, a low-grade pro-inflammatory state in the uterus is the prerequisite for normal embryo implantation. To achieve this, trophoblast cells in embryo and many immune cells that transfers to uterus from the peripheral blood secreted large amount of pro-inflammatory factors [23]. In the second trimester, the mother adapts to the presence of the fetus, and in order to create a suitable environment for the rapid growth and development of the fetus, the pro-inflammatory factors in the placenta significantly decreased and anti-inflammatory factors increased. In the third trimester, the pro-inflammatory factors in the placenta increased again to allow the fetus and placenta to delivery smoothly [3]. However, due to the limited samples of the included studies, this expression pattern of IL-15 needs to be further confirmed by high-quality research with a large sample, and molecular biology experiments would be required to confirm whether IL-15 is a pro-inflammatory cytokine of the placenta. Besides the expression pattern, we also determined the source of IL-15 in the placenta. Cells involved in secretory function in the placenta include cytotrophoblast cells, syncytiotrophoblast cells and macrophages [24]. Macrophages are mainly distributed at the center of the villi [25]. The immunofluorescence co-localization in our study showed that IL-15 was mainly distributed on the outside of the villi, which suggests that macrophages is not the origin of IL-15 in the placenta. Based on these results, we believe that the dominant source of IL-15 was the trophoblast cells in the placenta. Then we tested the expression of IL-15 in three trophoblast cell lines including HTR8-SVneo, JEG-3 and JAR, and found that all of them can express IL-15. Thus, we used them in subsequent experiments.

To adapt to the HG in GDM, many pro-inflammatory cytokines secreted by the placenta and adipose are increased significantly. For examples, IL-1β, TNF-α, IL-6, CRP and IL-18 were increased both in the placenta locally and the peripheral blood of GDM patients [7, 9, 26]. As an inflammatory cytokine, IL-15 expression was not only increased significantly in the placenta of GDM patients and in trophoblast cells under HG in vitro, but also was positively correlated with the concentration of blood glucose in our study. All these increased pro-inflammatory cytokines result from the HG-induced pro-inflammatory response by multiple pathways including: activation of Toll-like receptor 2 and Toll-like receptor 4, induction of oxidative stress and ERS, which ultimately promotes the progress of GDM itself [27, 28]. However, in our study, there was no significant difference in the level of IL-15 in the peripheral blood between GDM group and normoglycemic group. Given that IL-15 acts its biological function through autocrine and paracrine [15], we speculate that most of the IL-15 secreted by either trophoblasts or skeletal muscles may bind to IL-15R to form IL-15-IL-15R complex on the cell membrane, leading to the local depletion of IL-15 and the constant level of IL-15 in the peripheral blood.

As IL-15 is an active autocrine and paracrine cytokine, we speculated that IL-15 secreted by trophoblasts may have an impact in the trophoblast itself and contribute to the pathogenesis of GDM. Thus, we investigated the influence of IL-15 in cellular behavior of trophoblast cells by using the exogenous IL-15 and JAK inhibitors. In our results, IL-15 activated the JAK/STAT signaling pathway in trophoblast cells and ultimately promoted cell proliferation and up-regulated the capacity of invasion and tube formation in vitro. Mechanistically, IL-15 induces a conformational change of JAK forming a phosphorylated dimer, which further phosphorylates STATs resulting in the transcription of target gene. The target genes include PI3K-AKT and MAPK signaling pathway-associated molecules, which leads to different phenotypes of different cells [17]. In immune cells, previous studies demonstrated that IL-15-IL-15R complex stimulated NK cells and T cells proliferation and reduced apoptosis by activing the JAK/STAT signaling pathway [29]. In chronic muscle injury, IL-15 promoted the proliferation of fibro-adipogenic progenitor cells by activating the JAK/STAT signaling pathway, and further prevented the differentiation of fibro-adipogenic progenitor cells into the adipocytes in models both in vitro and in vivo [30]. IL-15 also promoted invasion of many tumor cells through activation of JAK/STAT signaling pathway, such as head and neck cancer cells, prostate cancer cells [31, 32]. In rheumatoid arthritis, IL-15 promoted the HUVEC tube formation which contributed to the disease progression [33]. In terms of trophoblasts, both the biological effects of IL-15 and the underlying mechanism were similar to other inflammatory factors including: IL-6, IL-11 and Epidermal Growth Factor [34]. Certainly, when JAK/STAT signaling pathway was activated by another factors, such as hypoxia and leptin, trophoblasts’ proliferation and invasion ability also increased significantly [35]. From these data and references, IL-15, in coordination with other molecules in GDM, may participate in the development of GDM by altering the proliferation and invasion ability of trophoblasts [36], and be responsible for the pathological change of placenta characterized by increased placental weight, number of villi and blood vessel density and thickened blood vessel wall [2].

Our results also showed that the expression of IL-15 in the placenta was negatively correlated with the weight of the newborns, which was consistent with previous studies. In obese adults, the concentration of IL-15 in the peripheral blood was much lower than the lean subjects, and it was negatively correlated with the mass of the trunk fat [37]. In mouse models of obesity, the white adipose volume and weight of mouse were reduced significantly through IL-15 administration or overexpression, while increased obviously in IL-15 knockdown models [38]. The role of IL-15 in regulations of both glucose and lipid metabolism may explain these observations. By activating PPARδ/α signaling pathway and alleviating ERS, IL-15, on the one hand, can promote the transportation and decomposition of free fatty acids. On the other hand, it can improve insulin sensitivity, decrease fasting blood glucose and serum insulin [13, 37, 39, 40]. This implies that IL-15 may play a positive role in the regulation of glucose and lipid metabolism. However, it has also been reported that IL-15 knockdown mouse showed much lower body weight, subcutaneous adipose volume and blood glucose levels compared to control mouse in HFD [41–43]. Therefore, the exact role of IL-15 in metabolic diseases still remains elusive, and further studies are needed.

## Conclusion

In conclusion, we explored variations of IL-15 in the placenta during pregnancy, and demonstrated that trophoblasts were the major source of IL-15 in maternal–fetal interface. IL-15 expression of GDM patients was observed upregulated in the placenta, not in peripheral blood. It was also positively correlated with blood glucose concentrations in OGTT, and negatively correlated weight of newborns. In vitro, IL-15 regulated the biological behaviors of trophoblasts through JAK/STAT signaling pathway. These findings provide a new direction for studying the pathophysiological changes of placenta in GDM patients.

## Materials and methods

### Participant selection and samples collection

This study was approved by the local ethic committee at Tongji Hospital. All the participants provided a written informed consent. The first (n = 3) and second (n = 3) trimester placental samples were obtained from pregnant women after legal termination due to psychosocial reasons. The third trimester placental and peripheral samples were obtained from normoglycemic pregnant women (n = 20) and GDM patients (n = 20). Women with GDM were diagnosed following the International Association of the Diabetes and Pregnancy Study Groups (IADPSG) guidelines; participants needed to obtain the OGTT between 24 and 28 of the gestational periods. Participants could be diagnosed with GDM if when fasting glucose level was above 5.1 mmol/L, and/or 1 h postprandial glucose level was above 10.0 mmol/L, and/or 2 h postprandial glucose level was above 8.5 mmol/L. These patients were treated through clinical intervention. Subjects were excluded from the study on the basis of having: multiple pregnancy; type 1 diabetes or diabetes mellitus before pregnancy; pre-eclampsia; chronic hypertension; renal disorder; a history of smoking more than five cigarettes per day during pregnancy.

All the placenta samples were collected and dissected immediately, then transported in liquid nitrogen and stored at − 80 °C or in paraformaldehyde until further analysis. Peripheral blood sample was extracted before delivery.

### Generation two new cell lines in normal glucose

HTR-8/SVneo and JAR were purchased from Servicebio company (Wuhan, China) and OBIO Technology company (Shanghai, China) respectively. JEG-3 was purchased from FuHeng biology company (Shanghai, China), which cultured in MEM medium (5.5 mmol/L glucose). HTR-8/SVneo and JAR were routinely cultured in RPMI-1640 medium with a glucose concentration of about 11.1 mmol/L, which was much higher than normal blood glucose (5.5 mmol/L), rendering that cells inappropriate for any glucose-related human diseases studies. Therefore, two new lines, HTR-8/SVneo-NG and JAR-NG, were generated by culturing cells in the physiological glucose concentration of 5.5 mmol/l in low-glucose DMEM medium. In order to ensure cells were totally adapted to the new environment, we used the cells after 20 passages and named them HTR-8/SVneo-NG and JAR-NG [44].

### Cell culture and treatment

To mimic HG condition, additional D-glucose (Sigma, Germany) was dissolved in normal culture medium (MEM and low-glucose DMEM medium) up to a final glucose concentration of 25 mmol/L. All trophoblast cell lines exposed to HG medium for 48 h represent a validated model of HG in vitro [28, 45].

Previous studied have demonstrated that IL-15 mainly active the JAK/STAT signaling pathway. To examine the effect of IL-15 on trophoblasts biological behaviors, we used Tofacitinib [46] and a recombinant human IL-15 to treat the cells. Cell proliferation was evaluated using a Cell Counting Kit 8 (HY-K0301, Medchem Express, USA) and a colony formation assay following the manufacture’s protocols. Colonies containing more than 50 cells were counted last. The trophoblasts were pretreatment with Tofacitinib and IL-15 mediums for 48 h. Subsequently, all the cells were plated in Transwell chambers (8.0 μm pore size, 3422, Corning, USA) coated with Matrigel (1.5 mg/mL) (354,234, Corning, USA) for 24 h. For tube formation, 60 uL Matrigel (10 mg/mL) was layered into 96-well plate first, then cells were seeded and incubated for 6 h. Images were captured using a microscope (Olympus BX53, Tokyo, Japan) in five random fields. Tube formation was assessed by the total tube length using Image J software.

### Single molecule array technology

Single molecule array (SiMoA) is an ultrasensitive technology that can detect proteins at sub-femtomolar concentrations (10^−16^ M) in blood, which is 1000 times higher in sensitivity compared to ELISA [47]. We used SiMoA technology to detect the level of IL-15 in human serum in collaboration with Novogene company (Beijing, China).

### RNA isolation and RT-PCR

Total RNA was extracted using TRIZOL reagent (Takara, Japan) and cDNA synthesized from 1ug RNA by using Takara reverse transcription kit (Takara, Japan) following the manufacturer's protocol. RT-PCR was carried out using the CFX96 Touch Real-time PCR Detection System (BIO-RAD, USA). The mRNA expression levels were normalized to the expression of β-actin housekeeping gene. Relative target gene expression was calculated using the 2−ΔΔCq method. The sequences of the primers used are as follows: β-actin upstream, 5′-TGGCACCCAGCACAATGAA-3′ and downstream, 5′-CTAAGTCATAGTCCGCCTAGAAGCA-3′. IL-15 upstream, 5′-AACAGAAGCCAACTGGGTGAATG-3′ and downstream, 5′-CTCCAAGAGAAAGCACTTCATTGC-3'.

### Western blot analysis

Placental samples and cells lysates were subjected to western blot analysis according to standard protocols. The membranes after blocking were incubated with a primary antibody overnight at 4 °C and subsequently incubated with horseradish peroxidase-conjugated secondary antibodies (Antgene, Wuhan, China). Primary antibodies include: ERK1/2 (ab17942, Abcam, Cambridge, UK), IL-15 (ab7213, Abcam, Cambridge, UK) and JAK/STAT pathway antibody sampler kit (97999 T, Cell signaling technology, Danvers, MA, USA). Blots were developed using an enhanced chemiluminescence substrate (Millipore, USA), and detection done using LAS-4000 (G: BOX, Gene company limited, Hong Kong, China).

### Immunohistochemistry and Immunofluorescence

Paraffin sections of formalin‐fixed samples were cut into 5 um thick sections and analyzed using an immunohistochemistry kit (Neobioscience, Wuhan, China). All sections were first incubated with IL-15 primary antibody at a 1:100 dilution (ab7213, Abcam, Cambridge, UK) at 4 °C overnight. The sections were then incubated with reagent B from the kit and finally, sections were stained with DAB working reagent for 2 to10 min depending on the degree of stain required and the cell nucleus stained with hematoxylin.

For immunofluorescence, all sections were first incubated with IL-15 primary antibody (ab7213, Abcam, Cambridge, UK) in combination with CK7 (YM3054, Immunoway, Beijing, China) or Vimentin (ab92547, Abcam, Cambridge, UK) or CD31 (1:150, ab134168, Abcam, UK) at 4 °C overnight. Then, the sections were incubated with a secondary antibody, Alexa Fluor 594 Donkey Anti rabbit IgG or Alexa Fluor 488 Donkey Anti mouse IgG (Antgene, Wuhan, China). The DNA dye-4′, 6-diamidino-2-phenylindole (DAPI), was used for nucleus staining. In the control experiments, primary antibodies were omitted. Images were captured using a fluorescence microscope (Olympus BX53, Tokyo, Japan) and the mean values of area-integrated optical densities analyzed using Image-Pro Plus 6.0 (Media Cybernetics, Denver, USA).

### Statistical analysis

Three independent repeats of all experiments were made. Statistical analysis was carried out using SPSS software 21 (SPSS Statistics Inc., Chicago, USA). Associations were analyzed using the Spearman correlation test. Results were showed as mean ± SDM. T-test was used for comparison of the mean of two groups whereas one way ANOVA was used for mean comparison of more than two groups. Normally distributed data was analyzed using an independent-samples t-test whereas abnormally distributed data was analyzed using the Kruskal–Wallis test followed by the Mann-Whitney*U* test. If Levene test for homogeneity demonstrates unequal variances among these the groups, P values calculation of Welch-corrected ANOVA was followed with a Games-Howell post-hoc-test. All graphs were generated using GraphPad Prism 7.0. A P value of less than 0.05 suggested that the difference was statistically significant.

## Data Availability

All data generated or analyzed during this study are included in this published article.

## Abbreviations

IL-15: Interleukin-15
GDM: Gestational diabetes mellitus
OGTT: 75G Oral Glucose Tolerance Test
T2DM: Type 2 diabetes mellitus
NF-κB: Nuclear factor-κB
PPAR: Peroxidase proliferator-activated receptor
AMPK: AMP-Activated protein kinase
GSK3: Glycogen synthase kinase 3
ERS: Endoplasmic reticulum stress
BAT: Brown adipose tissue
HUVEC: Human umbilical vein endothelial cells
SiMoA: Single molecule array
HG: High Glucose
