# Correction to: Upregulation of IL-15 in the placenta alters trophoblasts behavior contributing to gestational diabetes mellitus

**DOI:** 10.1186/s13578-021-00714-1

**Published:** 2021-12-13

**Authors:** Jiaqi Li, Yuan Li, Xuan Zhou, Lijie Wei, Jingyi Zhang, Shenglan Zhu, Huiting Zhang, Xuan Gao, Lali Mwamaka Sharifu, Shaoshuai Wang, Ling Xi, Ling Feng

**Affiliations:** National Clinical Research Center of Gynecology and Obstetrics, Tongji Hospital, Tongji Medical College, Huazhong University of Science and Technology, 1095 Jiefang Anv, Wuhan, 430030 Hubei China

## Correction to: Li et al. Cell Biosci (2021) 11:33 https://doi.org/10.1186/s13578-021-00533-4

In this article, there was a mistake in the Western blot photographs of p-JAK1 and p-JAK2 of JAR. The correct version of Figure 6 is given in this erratum (Fig. 6).

**Fig. 6:**
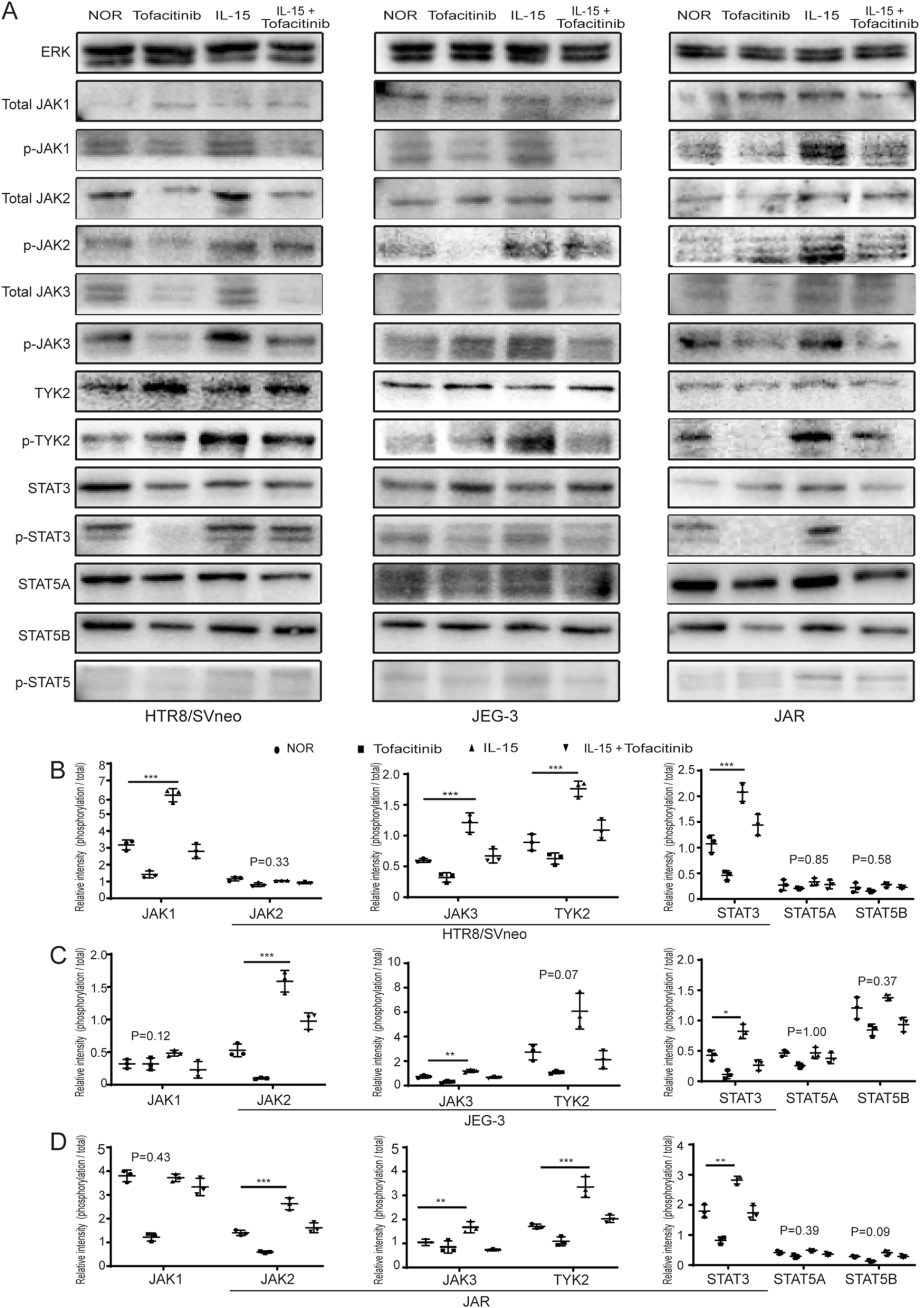
IL-15 activated the JAK/STAT signaling pathway in trophoblasts in vitro. **a** Representative of western blot showed the expression of proteins associated with JAK/STAT signaling pathways in different groups of HTR8/SVneo, JEG-3 and JAR. **b**–**d** The relative expression of phosphorylation/total protein in JAK/STAT signaling pathways in different groups of HTR8/SVneo, JEG-3 and JAR. *P < 0.05, **P < 0.01, ***P < 0.001

All the changes requested are implemented in this correction and the original article [1] has been corrected.
